# Distribution and determinants of functioning and disability in aged adults - results from the German KORA-Age study

**DOI:** 10.1186/1471-2458-13-137

**Published:** 2013-02-14

**Authors:** Ralf Strobl, Martin Müller, Rebecca Emeny, Annette Peters, Eva Grill

**Affiliations:** 1 Institute for Medical Information Processing, Biometrics and Epidemiology, Ludwig-Maximilians-Universität München, Munich, Germany; 2 Institute of Epidemiology II, Helmholtz-Zentrum München, Neuherberg, Germany

**Keywords:** Aged, Aged, 80 and over, Disability evaluation, Activities of daily living

## Abstract

**Background:**

Today industrialized countries face a burgeoning aged population. Thus, there is increasing attention on the functioning and disabilities of aged adults as potential determinants of autonomy and independent living. However, there are few representative findings on the prevalence and determinants of disability in aged persons in the German population.

The objective of our study is to examine the frequency, distribution and determinants of functioning and disability in aged persons and to assess the contribution of diseases to the prevalence of disability.

**Methods:**

Data originate from the MONICA/KORA study, a population-based epidemiological cohort. Survivors of the original cohorts who were 65 and older were examined by telephone interview in 2009. Disability was assessed with the Health Assessment Questionnaire Disability Index (HAQ-DI). Minimal disability was defined as HAQ-DI > 0. Logistic regression was used to adjust for potential confounders and additive regression to estimate the contribution of diseases to disability prevalence.

**Results:**

We analyzed a total of 4117 persons (51.2% female) with a mean age of 73.6 years (SD = 6.1). Minimal disability was present in 44.7% of all participants. Adjusted for age and diseases, disability was positively associated with female sex, BMI, low income, marital status, physical inactivity and poor nutritional status, but not with smoking and education. Problems with joint functions and eye diseases contributed most to disability prevalence in all age groups.

**Conclusions:**

In conclusion, this study could show that there are vulnerable subgroups of aged adults who should receive increased attention, specifically women, those with low income, those over 80, and persons with joint or eye diseases. Physical activity, obesity and malnutrition were identified as modifiable factors for future targeted interventions.

## Background

As in other industrialised countries, the German population is ageing. A decreasing number of births is paralleled with the aging of a large stratum of presently middle-aged people. In 2008, 5% of the German population was 80 or older; the estimated prevalence of those very old in 2060 is 14%, i.e. one seventh of the population will be 80 or older [1]. It is therefore of interest to know what extent of dependency and need for health care is to be expected, what choices and decisions must be made, and which kind of technologies might be needed [2].

The level of dependency is determined by functioning and disability, respectively. Disability denotes all impairments, activity limitations and participation restrictions [3] that are often operationalised by restrictions in activities of daily living and mobility. Conversely, functioning describes the positive aspects of these concepts.

Nevertheless, information on the exact prevalence of disability and the contribution of diseases to his prevalence is scarce for the German population [4]. Many German studies use information of nursing care level only as indicated by status according to German statutory long term care insurance. As an example, the Federal Bureau of Statistics indicates that 12% of persons aged 65 and above and 59.1% of the oldest old were in need of professional care [5].

Against this background, the German Federal Ministry of Education and Research (BMBF) initiated the “Health in Old Age” program to answer questions on successful aging and multimorbidity in Germany. The program consists of a total of six research consortia initially funded for a three year period (2008–2011). Among these, the KORA-Age study focuses on determinants and consequences of multimorbidity in the aged.

Known risk factors for disability other than age and sex include presence of cognitive impairment, depression, chronic diseases, multimorbidity, functional limitation in the legs, medication, visual impairment, physical environment and health-related life style like malnutrition, overweight, low physical activity level, smoking, alcohol intake, [6-8]. A recent literature review additionally analysed the contribution of frailty symptoms to disability in community-dwelling elderly people. Slow gait speed and low exercise level seemed to be the most important factors, followed by weight loss, low extremity and balance functions [9].

Conceptually, a person’s health status, as well as personal and contextual characteristics such as family support, influences his or her functioning. In old age, disability is not so much the result of a single disease but rather the effect of a complex simultaneous decrease in functioning of multiple physiologic systems [10]. This explains why older persons are very heterogeneous in regards to disability and why focussing on summary indices based on the number of health conditions does not reveal the true burden of disease on disability [11,12]. A rare disease may be very disabling on the individual but not on the population level, whereas highly prevalent diseases with small individual impact still contribute greatly to the societal burden of disease. Thus, an approach is warranted that combines prevalence of disease along with its impact on disability. Recently, methods have been proposed which deal with this in an appealing way [13,14].

The aim of this study was to present the prevalence of disability in a sample of the German population, and to examine the factors that are associated with disability. Specifically, we wanted to examine the contribution of health status, health-related lifestyle, sex, age, and socioeconomic status on disability and to estimate the disabling impact of chronic diseases.

## Methods

### Study design and participants

Data for this study were obtained from the KORA-Age cohort study. The KORA-Age project is carried out within the framework of KORA (Cooperative Health Research in the Region of Augsburg). KORA consists of four surveys S1-S4 which were based on a random sample of the population of Augsburg and the two surrounding counties Aichach-Friedberg and Augsburg [15]. The KORA-Age study is a cross-sectional follow-up including all participants of the MONICA/KORA Survey S1-S4 aged 65 years or older at the end of 2008, i.e. year of birth < 1942. Of a total of 17607 participants, 9197 fulfilled this criterion. 2734 of these 9197 persons died, 45 moved away and 427 refused to take part in the follow-up. The remaining 5991 persons have been contacted by a brief self-administered questionnaire. A postcard reminder was sent after 4 weeks and after another 4 weeks all non-responder were contacted by telephone. A total of 4565 persons completed this questionnaire either by mail or telephone. In addition, 5986 eligible persons have been contacted to participate in a telephone interview to which 4127 participated. More details about study design, sampling method and data collection of the MONICA/KORA surveys are reported elsewhere [15].

Interviews were performed by trained interviewers with medical background. If the participant was unable to conduct a telephone interview at first contact, either due to his or her mental or physical condition, a proxy telephone interview with a family member, friend or caregiver was carried out. All participants had given informed consent prior to their inclusion in the study or from the patient’s care giver in cases where the participant was unable to make an informed decision. Approval from the ethics committee of the Bavarian Medical Association was obtained.

### Measures: disability

Disability was assessed during the telephone interview with the Health Assessment Questionnaire Disability Index (HAQ-DI) [16]. The instrument consists of 20 questions in eight domains (dressing and grooming, hygiene, arising, reach, eating, grip, walking, and common daily activities), which can be answered on a scale from 0 (no difficulty), 1 (some difficulty), 2 (much difficulty) to 3 (unable to perform). The score of a domain is determined by the highest score in that domain. The HAQ-DI score is the mean of the eight domains. A HAQ-DI score of 0 corresponds to no disability while a HAQ-DI of 3 corresponds to severe disability. A more detailed description of the calculation can be found at [http://aramis.stanford.edu/HAQ.html]. The HAQ has been used with good success in a variety of settings and in the aged [17-19]. The German version had shown a good test-retest reliability, internal consistency and high criterion validity. The detailed wording of the German version and more information on the performance of the HAQ can be found in Bruehlmann et al. [20].

### Measures: covariates

All covariates were measured during telephone interview. Information on self-reported chronic disease (respiratory, gastrointestinal, heart, neurological, kidney and liver diseases, arthritis, cancer, stroke or diabetes mellitus) was collected with the Chaudhry questionnaire [21], supplemented by questions on fractures and eye diseases. Participants were asked: “Did you ever sustain a fracture?” and if yes, “What was the date (year) of the fracture?”. As fractures older than 5 years are unlikely to influence today’s functioning [22] we used the variable “Fracture in the last 5 years” in analysis.

Alcohol intake was measured as self-reported years of alcohol abstinence. Participants were asked: “Please indicate the year in which you stopped to drink alcoholic beverages, or since you drink very little alcohol?”. Based on this information they were classified as “abstinent” if they had stopped drinking or “not abstinent” else. Physical activity was classified using a modified short-version of the International Physical Activity Questionnaire (IPAQ) [23,24] as previously used in other MONICA/KORA studies [25]. Physical activity level was assessed at baseline using two four-category questions on the time spent per week on sport activities (including cycling) in summer and winter, respectively. Participants were classified as active if they participated for one or more hours in sports in summer and winter and as inactive else. Smoking habits were classified as never, former, occasional, regular smokers based on four questions:

(1) Have you smoked more than 100 cigarettes in your life?

(2) Do you smoke cigarettes at the moment?

(3) Do you smoke regularly or occasionally?

Participants were classified as “never” if they answered “no” to question (1), as “former” if they positively answered to question (1) but not to question (2), as “occasional” if they answered “occasionally” to question (3) and as “regular” if they answered “regularly”. Malnutrition was measured using a short version of SCREEN II (Seniors in the community: risk evaluation for eating and nutrition, Version II) [26]. SCREEN II is a 17-item questionnaire covering items such as unintentional and substantial weight loss, appetite, difficulties in swallowing and chewing, and cooking habits, yielding a score ranging from 0 to 64 with a lower score indicating increased risk.

For the purpose of the analyses, age was defined as age at interview and age groups were defined accordingly. BMI was calculated as body weight in kilograms divided by squared height in meters. Socioeconomic status was defined as years of education and per capita income in €1000. Information on education was obtained from the previous KORA study surveys S1-S4. Years of education were derived by combining information on the highest level of vocational training and of school graduation. Additionally, marital status, and gender were included into the analyses.

### Statistical analysis

We calculated mean and standard deviation for continuous variables and absolute and relative frequency for categorical variables stratified for persons with and without presence of disability. We used the Wilcoxon test to test for difference in continuous variables and the Pearson’s chi-squared test for categorical variables to test for difference in participants with and without disability. Following the literature [19], disability was defined as HAQ-DI >0. To visualize the relationship between HAQ-DI and age, stratified for gender, we used the lowess smoother which uses locally-weighted polynomial regression [27]. This yields a scatter plot of age versus quantiles of the HAQ-DI scores at the different ages.

#### Logistic regression

To investigate which factors were significantly associated with the presence of disability we used logistic regression. As independent variables we included diseases, lifestyle variables, socioeconomic status, age, gender and marital status. Selection of diseases was based on the Charlson Comorbidity Index. We added information on other chronic conditions which seemed relevant for assessing disability prevalence, namely fractures, eyes diseases and neurologic diseases. In addition, we included all measure on SES (education and income) and lifestyle variables (physical activity, smoking and alcohol) and sex, age and marital status. We calculated a logistic regression model including all of these variables as determinants of presence of disability. We used the c-value as a non-parametric measure of accuracy and predictive power [28]. The c-value varies between 0.5 and 1; the higher the c-value the better the model. Model fit was tested by the Hosmer-Lemeshow statistic, which should be non-significant (p > 0.05) to maintain the null hypothesis of adequate fit [29]. We tested for collinearity using the Variance Inflation Factor (VIF) and for logit-linearity using Box-Tidwell tests.

#### Attribution method

Prevalence of disability attributable to disease on a population level depends on both: the frequency of disease in the population and the disabling impact of the disease as estimated by a disease-specific rates in a multiple regression model. This concept is similar to the epidemiological concept of the population-attributable risk. To put this into context, while a rare but very disabling disease will have but a small impact on the burden of disability on the population level, a frequent disease with small impact on disability can attribute much to the burden. To estimate factor-specific disability prevalences we applied an additive hazards regression technique. This method allows to combine information on disease frequencies and disabling impact of a specific disease to calculate the disease-specific prevalence of disability [14]. The main idea behind this model is to divide the prevalence of disability into parts attributable to specific diseases or risk factors. Since many disabled persons will report more than one disease the attributable prevalence shows the contribution of one specific disease adjusted for co-morbidity. If a disabled person does not report any disease, this burden of disability will have to be attributed to the baseline risk of each person – so-called background risk – to incorporate the risk for developing disability due to unmeasured factors. In cases where a disabled person presents with one single disease, the burden of disease is partitioned into the contribution of disease and of background.

Thus, the additive regression model decomposes burden of disability into smaller parts that add up to total disability prevalence. For example, the risk for a person with diabetes and eye disease is decomposed into three different parts: the eye-specific risk, the diabetes-specific risk and the background risk. The sum gives the overall risk for disability for this person.

The model is specified as follows:

$$\eta =\alpha_{\alpha }+\sum_{d}\left(\gamma_{\alpha }\times \beta_{d}\cdot X_{d}\right)$$

with X_*d*_ representing the prevalence of disease, *β*_*d*_ the impact of each disease on disability and α_α_ the background risk for different age groups α. In the context of this model we assumed that disease-specific and background risk increase with age. To account for the age-specific impact of each disease the age and disease-specific effect is estimated as the product of an age effect which varies by age but not by disease γ_α_ sand the age-independent disease-specific effect *β*_*d*_. This Reduced Rank Regression is more parsimonious than assuming differing age effects per disease [30]. We also calculated a more complex Reduced Rank Regression which accounts for different age effects for each disease and present these result in an electronic supplement.

The overall risk can be transformed to a probability for disability *P*^*Disab*^ by applying the formula *P*^*Disab*^ = 1 − exp(−*η*). The sum of the probabilities for each disease across persons yields the number of disabled by this factor in the population.

Confidence intervals for each factor-specific disability prevalence were estimated by applying bootstrapping based on 1000 replicates [31]. Presence of disability was defined as a HAQ-DI score greater than zero in the attribution model.

We carried out all analyses and produced all figures with R 2.12.1 [32].

## Results

A total of 5991 participants of the S1-S4 surveys were contacted by postal health survey with a response rate of 76.2% resulting in 4565 completed questionnaires. Of these persons 4127 (90.4%) participated in a telephone interview. Due to missing values in more than 2 of the HAQ-DI domains, the sum score could be calculated for 4117 of these participants.

**Figure 1:**
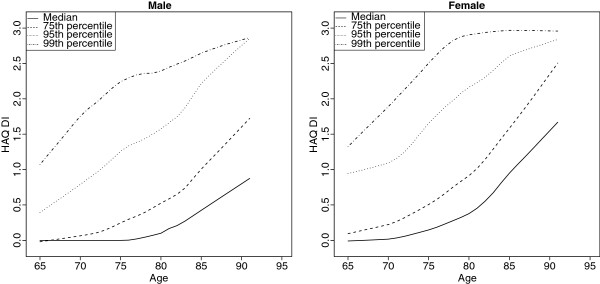
**Quantile curves of the association of disability and age.** Quantile curves of the association of disability and age. The curves were plotted by fitting lowess smoother on the percentile values

The 4117 participants had a mean age of 73.6 years (SD 6.1) and were 51.2% female. The mean HAQ-DI score was 0.32 with 44.7% of all participants presenting with any disability (HAQ-DI > 0) and 22.5% of all participants presenting with moderate or severe disability (HAQ-DI > = 0.5). Prevalence of disability was 27.6% in those aged 65 to 69, 45.4% in those aged 70 to 79 and 72.3% in those 80 years and older. Table 1 shows the characteristics of the sample population. Figure 1 shows the association of age and disability prevalence stratified by percentile of disability. Percentage of individuals with disability increased in a non-linear way by age, with a steep increase observed in participants older than 80. In all age groups, women presented with more severe disability than men. 20.1% of participants reported needing some form of assistance (25.4% women, 14.5% men). Most frequently, participants reported limitations in the domains reach (31.3%) and common daily activities (26.4%), least frequently in the domains grip (8.5%) and eating (12.1%) (see Additional file 1). Adjusted for age and diseases (see Table 2), disability was positively associated with female sex, BMI, low income, marital status, physical inactivity and poor nutritional status, but not with smoking and education. Women had a 2.5 fold increased risk for disability. Neurologic diseases (OR = 2.98), stroke (OR = 2.68) and diseases of the joint (OR = 2.36) had the strongest negative effect on disability. We could not a show an effect of liver disease and cancer. There was no significant difference in disability among smokers and non-smokers. Model fit was adequate (Hosmer-Lemeshow p-value = 0. 2436, c-value = 0.81).

**Table 1:** Characteristics of participants stratified by disability status

		**n**	**Total**	**No disability**	**Any disability**	**p-value**
Age at examination		4117	73.56 (6.09)	71.65 (5.06)	75.92 (6.42)	< 0.0001
Sex	Female	4117	2109 (51.2%)	974 (42.8%)	1135 (61.7%)	< 0.0001
Marital Status	unmarried	4062	148 (3.6%)	69 (3.1%)	79 (4.4%)	< 0.0001
	divorced		229 (5.6%)	121 (5.4%)	108 (6%)	
	widowed		954 (23.5%)	387 (17.2%)	567 (31.3%)	
	married		2731 (67.2%)	1676 (74.4%)	1055 (58.3%)	
Education (per year)		4116	10.84 (2.45)	11.2 (2.59)	10.39 (2.18)	< 0.0001
Per Capita Income in €1000		3830	1.11 (0.53)	1.16 (0.56)	1.04 (0.49)	< 0.0001
Smoking	never	4113	2242 (54.5%)	1197 (52.6%)	1045 (56.9%)	0.0047
	former		1611 (39.2%)	919 (40.4%)	692 (37.7%)	
	occasional		27 (0.7%)	12 (0.5%)	15 (0.8%)	
	regular		233 (5.7%)	148 (6.5%)	85 (4.6%)	
Alcohol abstinence	Yes	4088	649 (15.9%)	247 (10.9%)	402 (22.1%)	< 0.0001
Malnutrition score		4117	38.27 (5.47)	39.5 (4.71)	36.74 (5.93)	< 0.0001
Physical activity	Active	4117	2290 (55.6%)	1542 (67.7%)	748 (40.7%)	< 0.0001
BMI		4081	27.41 (4.18)	26.97 (3.69)	27.96 (4.67)	< 0.0001
**Diseases**						
Pulmonal disease	Yes	4117	433 (10.5%)	161 (7.1%)	272 (14.8%)	< 0.0001
Joint disease	Yes	4117	676 (16.4%)	239 (10.5%)	437 (23.8%)	< 0.0001
Gastrointestinal disease	Yes	4117	361 (8.8%)	154 (6.8%)	207 (11.2%)	< 0.0001
Heart disease	Yes	4117	1087 (26.4%)	468 (20.6%)	619 (33.6%)	< 0.0001
Stroke	Yes	4112	283 (6.9%)	84 (3.7%)	199 (10.8%)	< 0.0001
Kidney disease	Yes	4117	168 (4.1%)	65 (2.9%)	103 (5.6%)	< 0.0001
Liver disease	Yes	4117	95 (2.3%)	43 (1.9%)	52 (2.8%)	0.0591
Cancer	Yes	4105	178 (4.3%)	89 (3.9%)	89 (4.9%)	0.1625
Diabetes	Yes	4110	707 (17.2%)	301 (13.2%)	406 (22.1%)	< 0.0001
Fracture in last 5 years	Yes	4088	420 (10.3%)	163 (7.2%)	257 (14.1%)	< 0.0001
Neurologic Disease	Yes	4117	142 (3.4%)	39 (1.7%)	103 (5.6%)	< 0.0001
Eye Disease	Yes	4116	1585 (38.5%)	703 (30.9%)	882 (48%)	< 0.0001

No disability (HAQ-DI = 0) vs. Any disability (HAQ-DI > 0): For continuous variables we report mean and standard deviation in parentheses. For categorical variables we report absolute frequencies and column-wise relative frequencies. We used Wilcoxon rank-sum test to test for difference in continuous variables and Pearson's chi-squared test for categorical variables.

**Table 2:** Results of multivariable logistic regression modelling the association with presence of any disability

**Variables**	**Odds ratio**	**95% CI**
**Age at Examination**	1.12	[1.11; 1.14]
**Sex (ref = male)**	2.49	[2.06; 3.02]
**Marital Status (ref = unmarried)**		
divorced	0.61	[0.36; 1.03]
widowed	0.57	[0.37; 0.89]
married	0.58	[0.38; 0.89]
**Education (per year)**	0.98	[0.94; 1.02]
**Per Capita Income in €1000**	0.70	[0.58; 0.83]
**Smoking (ref = never)**		
former	1.16	[0.97; 1.38]
occasional	2.44	[0.96; 6.28]
regular	0.87	[0.60; 1.25]
**Alcohol abstinence (ref = not abstinent)**	1.11	[0.89; 1.39]
**Malnutrition index**	0.93	[0.92; 0.95]
**Physical Activity (ref = not active)**	0.55	[0.47; 0.65]
**Body Mass Index**	1.07	[1.05; 1.09]
**Diseases**		
Pulmonal disease (ref = no)	1.63	[1.26; 2.11]
Joint disease (ref = no)	2.36	[1.91; 2.91]
Gastrointestinal disease (ref = no)	1.52	[1.16; 2.00]
Heart disease (ref = no)	1.31	[1.09; 1.56]
Stroke (ref = no)	2.68	[1.93; 3.74]
Kidney disease (ref = no)	1.52	[1.00; 2.33]
Liver disease (ref = no)	1.06	[0.62; 1.82]
Cancer (ref = no)	1.38	[0.95; 2.00]
Diabetes (ref = no)	1.28	[1.03; 1.57]
Fracture in last 5 years (ref = no)	1.44	[1.11; 1.88]
Neurologic Disease (ref = no)	2.98	[1.90; 4.75]
Eye Disease (ref = no)	1.23	[1.04; 1.45]

Table 3 shows observed frequencies of disease and disability prevalences for persons stratified by age group. Disability prevalence varied from 0.28 to 0.55 for participants aged 65 to 69, from 0.48 to 0.73 for participants aged 70 to 79 and from 0.77 to 0.88 for participants older than 80. In each age group disability prevalence was highest for participants with neurological diseases.

**Table 3:** Prevalence of disability per disease and age group

	**Total (n = 4077)**	**65-69 (n = 1333)**	**70-79 (n = 1987)**	**> = 80 (n = 757)**
	**n (% disabled)**	**n (% disabled)**	**n (% disabled)**	**n (% disabled)**
No disease	988 (24.5%)	479 (15.9%)	425 (28.7%)	84 (52.4%)
Pulmonal disease	425 (62.8%)	117 (43.6%)	217 (64.5%)	91 (83.5%)
Joint disease	667 (64.5%)	187 (55.1%)	338 (62.7%)	142 (81.0%)
Gastrointestinal disease	357 (57.1%)	107 (34.6%)	183 (60.7%)	67 (83.6%)
Heart disease	1072 (56.6%)	234 (40.6%)	553 (52.6%)	285 (77.5%)
Stroke	278 (69.8%)	54 (50.0%)	141 (70.9%)	83 (80.7%)
Kidney disease	166 (60.8%)	39 (28.2%)	90 (64.4%)	37 (86.5%)
Liver disease	95 (54.7%)	33 (45.5%)	46 (52.2%)	16 (81.2%)
Cancer	178 (50.0%)	58 (32.8%)	83 (48.2%)	37 (81.1%)
Diabetes	702 (57.1%)	178 (35.4%)	363 (59.0%)	161 (77.0%)
Fracture in last 5 years	419 (61.1%)	112 (37.5%)	212 (61.8%)	95 (87.4%)
Neurologic Disease	136 (72.1%)	33 (54.5%)	70 (72.9%)	33 (87.9%)
Eye Disease	1568 (55.4%)	314 (36.6%)	773 (49.8%)	481 (76.5%)

In each age group joint and eye diseases attributed most to the burden of disability on the population level. Disability prevalence attributable to joint disease varied from 12% for the age group under 70 to 7.2% for the persons aged 80 and older. Attributable prevalence of eye disease varied from 5.6% to 7.3% for the oldest. Although neurologic disease was the health condition with the highest impact on disability (OR = 2.98), only a small part of the burden of disability could be attributed to neurological disease on the population level. Prevalence of disability attributable to background risk was 51% in those aged 65 to 69, 50% in those aged 70 to 79 and increased to 61% in those 80 years and older (see Table 4).

**Table 4:** Disease-specific attributable prevalence of disability in percent points as estimated by the attribution method

	**65 - 69**		**70 - 79**		**> = 80**	
	**%**	**95% CI**	**%**	**95% CI**	**%**	**95% CI**
Background	51.2	[41.5; 60.4]	49.8	[43.5; 56.1]	61.3	[49.7; 72.3]
Pulmonal disease	5.1	[3.2; 7.4]	4.8	[2.9; 6.7]	3.2	[1.8; 4.9]
Joint disease	12.0	[8.5; 15.8]	10.8	[8.5; 13.2]	7.2	[4.9; 9.7]
Gastrointestinal disease	3.5	[1.8; 5.4]	3.0	[1.5; 4.8]	1.8	[0.8; 3.0]
Heart disease	5.0	[2.6; 7.9]	6.1	[3.4; 8.7]	5.1	[2.5; 8.0]
Stroke	3.2	[1.9; 4.7]	4.2	[2.7; 6.1]	4.0	[2.5; 5.9]
Kidney disease	0.7	[−0.5; 1.8]	0.8	[−0.4; 2.1]	0.5	[−0.3; 1.5]
Liver disease	0.5	[−0.4; 1.7]	0.4	[−0.3; 1.1]	0.2	[−0.2; 0.7]
Cancer	0.9	[−0.3; 2.3]	0.6	[−0.3; 1.6]	0.4	[−0.2; 1.2]
Diabetes	4.8	[2.5; 7.3]	5.0	[2.4; 7.7]	3.6	[1.8; 5.6]
Fracture in last 5 years	4.9	[3.1; 7.1]	4.6	[2.9; 6.4]	3.3	[1.7; 5.2]
Neurologic Disease	2.5	[1.3; 4.1]	2.6	[1.6; 3.8]	2.0	[1.0; 3.2]
Eye Disease	5.6	[2.7; 9.0]	7.2	[3.9; 10.7]	7.3	[3.4; 12.3]

We also calculated an attribution model with different age effects for each disease. This complex model yielded negative probabilities for kidney disease, which has to be removed from the equation. This can happen if the corresponding disease has a low prevalence or almost no impact on disability. We present the result of this model in an electronic supplement (see Additional file 2).

## Discussion

This study examined prevalence of disability and the disabling impact of risk factors and disease in a sample of the German population of age 65 and above. Minimal disability was highly prevalent in all age groups. Aging was associated with increasing prevalence of disability, with a steep increase in participants over 80. Female sex, lower per capita income, physical inactivity and malnutrition were factors significantly associated with disability when adjusting for age and disease. Not surprisingly, when analyzing the specific contribution of single diseases on disability prevalence on the population level we found that disability attributed to causes other than the diseases included in the analysis increased with age. Stroke and neurological diseases were strongly associated with disability, but joint diseases and eye disease contributed most to the burden of disability in this population.

Direct comparisons of prevalence of disability between studies are difficult because of the multitude of measures applied, and because of diverse populations and their differing living conditions. A study based on the LEILA 75+ survey in Leipzig observed a disability prevalence of 63.6% for community-dwelling persons aged 75 or above, but did not analyse prevalence of disability for other age groups [33]. The observed increase of disability prevalence with age, stratified by sex, in our study is consistent to findings of other studies [19,34]. A report of the German health reporting system based on the socio-economic panel also observed a sudden increased need for personal assistance at home for persons aged 80 or older [5].

However, sample sizes, specifically in age groups over 80, are usually small, thus making evidence in that age group inconclusive. Our study, with a sample size of 780 participants over 80, showed that disability increased in a curvilinear way with a steeper onset in the 8th life decade. A study in Netherlands included a similar number of persons in this age group, but observed a lower prevalence of disability for both sexes: 20% for men and 37% for women. A part of this difference can be explained by the varying conceptualisation of disability [13].

Also, in line with the literature [34-36], we found that prevalence of disability was higher in women across all age strata, levels of severity, and domains of disability. Sex differences in disability are often explained by co-morbidity and greater female longevity. It is argued that social and health related issues largely contribute to the higher prevalence of disability in women [37]. However, the association of sex and disability remained relevant in our study even when controlling for major health conditions and even when stratifying for age. Additionally, we found that differences were more pronounced in locomotor functions such as reach, grip, and hygiene, indicating that disability might be associated with general fitness and strength as a consequence of an inactive lifestyle (see Additional file 1). Since more women than men reported living alone in our study (41.5% vs. 14.9%) lack of assistance may have contributed to this finding. Indeed, more women than men reported needing assistance.

We could confirm the association of low socioeconomic status and disability at old age in our study. This association is well known from the literature [38]. Other studies have also shown that lower socioeconomic status increases the risk for physiological impairment, i.e. factors predisposing disability, such as muscle strength, range of joint motion and visual acuity [39].

The impact of physical activity on health and mortality is conclusive [40]. As was shown before the association of physical activity and disability persisted in our study, even after controlling for diseases [41].

The attribution method revealed joint diseases as being the strongest contributing factor to disability prevalence. A similar result was obtained in the study by Klijs et al. [13]. We could additionally show that diseases of the eye explained about 10% of the prevalence of disability. This result confirms findings from previous study on the role of eye on functional ability in the aged [42,43].

Increasing prevalence of disability by age, even in disease-free individuals, can be explained by increasing frailty. This is likely to be associated with decreasing muscle strength, decreased radius of activity and reduced cardiopulmonary fitness [44-47]. Joint disease and fractures contributed most to the burden of disability by being frequent diseases with a strong association to disability. This is in line with recent findings reporting musculoskeletal disease as the main contributors of disability [13]. Additionally, we could show in our study that diseases of the eye explained about 10% of the prevalence of disability. This result confirms findings from previous study on the role of vision on functional ability [42,43]. The results of our study might be promising because fractures as a consequence of falls are known contributors of disability [48] and can be prevented. Joint disease can be modified by exercise and weight control. Vision is also amenable to intervention. Nevertheless, more detailed data of the nature of eye and joint disease is needed to inform on the appropriate measures for intervention. .

We acknowledge several limitations in our study. Due to the observational, cross-sectional study design, causal associations could not be examined. Thus, disability may not only be a consequence of a disease, but also be a cause for a disease [3]. In situations like this where variables show complex interactions, reverse causation cannot be ruled out. To give an example, the true nature of the interaction of education, BMI and disability has to be examined more closely.

Additionally, information was collected by personal or proxy interview which is, in theory, prone to information bias. However, previous studies have shown that self report of health conditions in this study setting was reliable [49].

We also acknowledge that the sample might not be entirely representative of the general population of aged adults due to the exclusion of individuals who either chose not to participate because of disabling conditions or for whom no proxy information was available if they did participate. The issue of non-participation in one of the baseline surveys has been studied in detail before [50]. It was shown that non-participant include a higher fraction of persons with worse health and that severely impaired persons are less likely to participate in our study. As a result, our study may underestimate the true prevalence of disability and consequently also the true impact of disease on disability. Furthermore, the city of Augsburg and surroundings is not representative for Germany in terms of socio-economic status and deprivation [51].

We used years of education instead of the highest level of education, as this variable combines information on both vocational training and school education. This allowed us to differentiate between those with minimal school education and those with additional vocational training.

The attribution model itself also has several shortcomings and assumptions that need to be considered. A basic assumption of the model is that the distribution of diseases does not change over time. Moreover, disease and background morbidity are assumed to act as independently competing causes for disability that add up to the total risk. Also, it is assumed that all persons belonging to an age group have the same background disability risk.

## Conclusions

In conclusion, this study could show that there are vulnerable subgroups of aged adults who should receive increased attention, specifically women, those with low income, those over 80, and persons with joint or eye diseases. Physical activity, obesity and malnutrition were identified as modifiable factors for future targeted interventions.

## Competing interests

The authors declare that they have no competing interests.

## Authors’ contributions

RS and EG designed and conceptualization of the study. RS conducted the statistical analysis; EG supervised the study; all authors contributed to the analysis and writing, revised the manuscript critically for important intellectual content and approved the final manuscript. All authors read and approved the final manuscript.

## Pre-publication history

The pre-publication history for this paper can be accessed here:

http://www.biomedcentral.com/1471-2458/13/137/prepub

## Supplementary Material

Additional file 1 **Sex-specific percentages of HAQ domains.** No disability (HAQ-DI=0), small disability (HAQ-DI: 0 – 0.5), moderate disability (HAQ-DI: 0.5 – 1), severe disability (HAQ-DI: 1 - 3). Click here for file

Additional file 2 **Disease-specific prevalence of disability with disease-specific age differences.** Disease-specific attributable prevalence of disability in percent points accounts for different age effects for each disease. The algorithm yielded a negative estimate for the estimation of kidney disease. This variable was excluded from the final model to yield sensible estimates. Click here for file
