# Supplementary material for: Distribution and determinants of functioning and disability in aged adults - results from the German KORA-Age study
Source: BMC Public Health. 2013 Feb 14;13:137. doi: 10.1186/1471-2458-13-137 (PMC3635873; doi:10.1186/1471-2458-13-137)
Supplement: Additional file 1 — Sex-specific percentages of HAQ domains. No disability (HAQ-DI=0), small disability (HAQ-DI: 0 – 0.5), moderate disability (HAQ-DI: 0.5 – 1), severe disability (HAQ-DI: 1 - 3). [file 1471-2458-13-137-S1.doc]

Additional File 1: Sex-specific percentages of HAQ domains. No disability (HAQ-DI=0), small disability (HAQ-DI: 0 – 0.5), moderate disability (HAQ-DI: 0.5 – 1), severe disability (HAQ-DI: 1 - 3)

| **Sex** | n | **%No Disability** | **%Small Disability** | **%Moderate Disability** | **%Severe Disability** |
| --- | --- | --- | --- | --- | --- |
| **Dressing and Grooming** | | | | | |
| **Women** | 2110 | 83.6 | 9.3 | 2.7 | 4.4 |
| **Men** | 2010 | 85.6 | 9.1 | 2.8 | 2.5 |
| **Hygiene** | | | | | |
| **Women** | 2109 | 71.7 | 10.9 | 7.2 | 10.1 |
| **Men** | 2008 | 83.9 | 7.3 | 4.2 | 4.6 |
| **Arising** | | | | | |
| **Women** | 2110 | 75.4 | 18.9 | 3.1 | 2.6 |
| **Men** | 2009 | 83.3 | 13.0 | 2.2 | 1.4 |
| **Reach** | | | | | |
| **Women** | 2109 | 61.8 | 21.7 | 6.8 | 9.7 |
| **Men** | 2007 | 77.9 | 13.9 | 4.3 | 3.9 |
| **Eating** | | | | | |
| **Women** | 2110 | 85.0 | 9.7 | 2.8 | 2.6 |
| **Men** | 2009 | 91.0 | 5.1 | 2.1 | 1.7 |
| **Grip** | | | | | |
| **Women** | 2109 | 88.8 | 6.6 | 1.9 | 2.7 |
| **Men** | 2008 | 94.4 | 3.4 | 1.0 | 1.2 |
| **Walking** | | | | | |
| **Women** | 2108 | 76.3 | 14.1 | 6.6 | 2.9 |
| **Men** | 2009 | 83.4 | 9.7 | 5.2 | 1.7 |
| **Common Daily Activities** | | | | | |
| **Women** | 2109 | 68.3 | 17.2 | 6.9 | 7.6 |
| **Men** | 2008 | 79.2 | 11.8 | 4.1 | 4.9 |
