# Supplementary material for: Distribution and determinants of functioning and disability in aged adults - results from the German KORA-Age study
Source: BMC Public Health. 2013 Feb 14;13:137. doi: 10.1186/1471-2458-13-137 (PMC3635873; doi:10.1186/1471-2458-13-137)
Supplement: Additional file 2 — Disease-specific prevalence of disability with disease-specific age differences. Disease-specific attributable prevalence of disability in percent points accounts for different age effects for each disease. The algorithm yielded a negative estimate for the estimation of kidney disease. This variable was excluded from the final model to yield sensible estimates. [file 1471-2458-13-137-S2.doc]

## Table 1 - Disease-specific prevalence of disability with disease-specific age differences.

Disease-specific attributable prevalence of disability in percent points accounts for different age effects for each disease. The algorithm yielded a negative estimate for the estimation of kidney disease. This variable was excluded from the final model to yield sensible estimates.

|  | **65 - 69** | | **70 - 79** | | **>= 80** | |
| --- | --- | --- | --- | --- | --- | --- |
|  | **%** | **95% CI** | **%** | **95% CI** | **%** | **95% CI** |
| Background | 50.1 | [40.9; 60.5] | 51.7 | [44.6; 58.5] | 62.1 | [47.5; 73.3] |
| Pulmonal disease | 3.4 | [-0.1; 7.2] | 5.6 | [3.2; 7.7] | 3.2 | [1.0; 5.9] |
| Joint disease | 16.6 | [10.3; 21.7] | 8.8 | [6.3; 12.4] | 7.0 | [1.5; 10.1] |
| Gastrointestinal disease | 1.5 | [-1.0; 4.9] | 3.8 | [1.8; 5.7] | 1.8 | [0.4; 4.5] |
| Heart disease | 8.5 | [3.4; 13.0] | 4.3 | [0.7; 8.1] | 5.0 | [-0.9; 10.5] |
| Stroke | 2.5 | [0.3; 5.2] | 4.7 | [2.8; 6.8] | 4.0 | [0.0; 5.8] |
| Kidney disease | - | - | - | - | - | - |
| Liver disease | 1.2 | [-0.4; 3.0] | 0.1 | [-0.7; 1.0] | 0.2 | [-0.6; 1.0] |
| Cancer | 0.9 | [-1; 3.1.0] | 0.7 | [-0.5; 1.8] | 0.5 | [-0.5; 2.0] |
| Diabetes | 0.8 | [-3.0; 6.7] | 7.1 | [3.7; 10.3] | 3.7 | [-1.9; 6.9] |
| Fracture in last 5 years | 2.9 | [-0.6; 7.1] | 5.6 | [2.9; 7.4] | 3.4 | [1.8; 7.9] |
| Neurologic Disease | 2.3 | [0.7; 4.5] | 2.8 | [1.3; 4.1] | 2.0 | [0.7; 3.4] |
| Eye Disease | 9.3 | [1.9; 14.8] | 4.9 | [-0.1; 9.8] | 7.0 | [2.0; 20.7] |
